# Microallopatry Caused Strong Diversification in *Buthus* scorpions (Scorpiones: Buthidae) in the Atlas Mountains (NW Africa)

**DOI:** 10.1371/journal.pone.0029403

**Published:** 2012-02-27

**Authors:** Jan C. Habel, Martin Husemann, Thomas Schmitt, Frank E. Zachos, Ann-Christin Honnen, Britt Petersen, Aristeidis Parmakelis, Iasmi Stathi

**Affiliations:** 1 Natural History Museum Luxembourg, Invertebrate Biology, Luxembourg; 2 Baylor University, Biology Department, Waco, Texas, United States of America; 3 Trier University, Department of Biogeography, Trier, Germany; 4 Natural History Museum Vienna, Vienna, Austria; 5 Zoological Institute, Christian-Albrechts-University, Kiel, Germany; 6 Institute of Clinical Molecular Biology, University Hospital SH Campus Kiel, Kiel, Germany; 7 National and Kapodistrian University of Athens, Department of Ecology and Taxonomy, Athens, Greece; 8 Natural History Museum of Crete, University of Crete, Heraklion, Crete, Greece; University of York, United Kingdom

## Abstract

The immense biodiversity of the Atlas Mountains in North Africa might be the result of high rates of microallopatry caused by mountain barriers surpassing 4000 meters leading to patchy habitat distributions. We test the influence of geographic structures on the phylogenetic patterns among *Buthus* scorpions using mtDNA sequences. We sampled 91 individuals of the genus *Buthus* from 51 locations scattered around the Atlas Mountains (Antiatlas, High Atlas, Middle Atlas and Jebel Sahro). We sequenced 452 bp of the Cytochrome Oxidase I gene which proved to be highly variable within and among *Buthus* species. Our phylogenetic analysis yielded 12 distinct genetic groups one of which comprised three subgroups mostly in accordance with the orographic structure of the mountain systems. Main clades overlap with each other, while subclades are distributed parapatrically. Geographic structures likely acted as long-term barriers among populations causing restriction of gene flow and allowing for strong genetic differentiation. Thus, genetic structure and geographical distribution of genetic (sub)clusters follow the classical theory of allopatric differentiation where distinct groups evolve without range overlap until reproductive isolation and ecological differentiation has built up. Philopatry and low dispersal ability of *Buthus* scorpions are the likely causes for the observed strong genetic differentiation at this small geographic scale.

## Introduction

The Mediterranean region comprises a highly diverse geographic area characterized by a variety of peninsulas and islands. This underlying geographic structure is reflected in biogeographical patterns of the region already described by de Lattin (1949) [1] who distinguished nine Mediterranean sub-centres. These early hypotheses have largely been confirmed by genetic analyses [2]. However, recent molecular data support even more fine-scaled patterns within these sub-centres; such patterns are especially known from the Iberian Peninsula e.g. [3], peninsular Italy e.g. [4] and the Balkans [5]. While the European part of the Mediterranean is biogeographically relatively well understood, the Maghreb still remains largely unstudied [6]. Therefore, the question emerges whether similar genetic substructures also exist in NW Africa.

De Lattin (1949) [1] suggested that the faunas of the Maghreb differ north and south of the High Atlas; this pattern is well reflected in the geographic distributions of many species e.g. [7]. On the contrary, other genetic studies revealed an east-west split along the Maghreb coast [8]. Furthermore, no genetic differentiation from Morocco to Tunisia was observed in the allozyme patterns of two butterfly species [9]. Nevertheless, all these genetic patterns are much simpler compared to findings in southern Europe. This is particularly astonishing considering the high orographic diversity of the Maghreb in general and the Atlas Mountains in particular. Yet, only few studies have addressed the phylo- or biogeographic patterns within the Atlas Mountains, although this area comprises an excellent region to study allopatric differentiation.

In order to test for the influence of orographic structures on differentiation and speciation processes, we selected the scorpion genus *Buthus*, which has undergone extensive speciation within NW Africa [10], as a model system. *Buthus* scorpions are widely distributed over large parts of Africa and show a circum-Mediterranean stronghold including the Mediterranean islands, with extraordinarily high diversity in North Africa [10]–[14]. Yet, molecular studies showed that diversity may still be underestimated and additional cryptic species may be abundant [14]. Most buthid scorpions are strictly territorial and philopatric and have low dispersal abilities. Consequently, individuals have limited home ranges which generally are restricted to small areas around their burrows [15]. Strong genetic differentiation has previously been observed for scorpion species and was probably triggered by barriers such as high elevations, steep valleys or sea barriers [16]–[19] in combination with low dispersal ability of the organisms [20]. In addition to being extremely limited, dispersal is also highly asymmetrical in these organisms, with male scorpions dispersing more widely. This apparently is a function of mate, prey and shelter search, where males exhibit a much more opportunistic locomotive behaviour, whereas females stay in their shelter and forage mainly close to the entrance of their burrows (“doorkeeping” strategy) [21], [22].

For this study we collected a total of 91 individuals belonging to 4 described *Buthus* species from 51 locations over major parts of the Antiatlas, High Atlas, Middle Atlas and Jebel Sahro during spring 2008 and 2009. Three specimens of the related genus *Androctonus* from two locations served as outgroup. We analysed a fragment of the mitochondrial Cytochrome Oxidase I (*COI*) gene, which has been shown to be highly informative at species level and consequently is one of the most commonly used markers for phylogenetic and phylogeographic studies. Due to its high potential for species identification it is often chosen as the barcoding gene in zoological studies. Here we use its properties to address the following questions:

- Does genetic variation within *Buthus* exhibit geographic structuring?
- Do genetic structures follow any geographical and/or evolutionary pattern?
- Are genetic lineages in accordance with current taxonomy?

## Results

We generated 94 sequences (including three outgroup specimens) with a total length of 452 base pairs. One sequence from *Androctonus mauritanicus* (JF820097) was obtained from genbank as an additional outgroup. Details about analyzed individuals, including genbank accession numbers, are given in Table 1. Out of the 452 sites analysed, 135 were polymorphic (29.9%) of which 127 were parsimony informative sites (4 sites had missing data). Within the ingroup we recovered 69 haplotype with a haplotypes diversity of Hd = 0.993 (SD = 0.003) and a nucleotide diversity of Pi = 0.0916 (SD = 0.0018). The average number of nucleotide differences was k = 41.138. Fst estimates among groups obtained from phylogenetic analyses were overall high and mostly significant (Table 2; non significant values are due to low sample sizes in some groups).

**Table 1:** Sampling localities of all studied *Buthus* populations.

Group	Locality	Site	Coordinates(N; W)	Species	Date of sampling	Specimen ID/##	Genbank accession#
**A**	Tamensourt	1	31.43; 8.01	*B. elmoutaouakili*	13-III-2009	9-80-5	JN885948
**B**	Chichoaua	2	31.27; 8.47	*B. elmoutaouakili*	13-III-2009	9-79-1	JN832012
						9-79-2	JN832013
**C**	Argane	3	30.55; 9.03	*B. elmoutaouakili*	12-III-2009	9-78-2	JN832008
						9-78-3	JN832009
**D**	Ameskrout	4	30.37; 9.20	*B. elmoutaouakili*	12-III-2009	9-77-1	JN832003
						9-77-2	JN832004
	Agadir	5	30.28; 9.18	*B. elmoutaouakili*	12-III-2009	9-74-4	JN831999
						9-74-6	JN832000
**E**	Tiznit	6	29.18; 9.45	*B. elmoutaouakili*	24-II-2009	9-13-1	JN831992
						9-13-2	JN831993
	Et Tnine	7	29.42; 9.16	*B. elmoutaouakili*	11-V-2008	8-6-1	JN831971
						8-6-2	JN831972
	Tighermi	8	29.32; 9.20	*B. elmoutaouakili*	24-II-2009	9-10-3	JN831983
						9-10-5	JN831984
	Onafka	9	29.24; 9.15	*B.* spec.	24-II-2009	9-12-1	JN831989
						9-12-2	JN831990
	Izerbi	10	29.20; 9.04	*B.* spec.	24-II-2009	9-11-1	JN831986
						9-11-2	JN831987
**F**	Ait Saha	11	30.06; 9.12	*B. elmoutaouakili*	23-II-2009	9-6-1	JN831995
						9-6-3	JN831996
	Tiz-n-Test	12	30.52; 8.23	*B.* spec.	12-V-2008	8-10-1	JN831974
						8-10-2	JN831975
	W Tassoumate	13	30.30; 8.34	*B. elmoutaouakili*	11-III-2009	9-73-2	JN832029
						9-73-3	JN832030
	Ait Aissa	14	30.18; 8.31	*Buthus* spec.	22-II-2009	9-3-1	JN832016
						9-3-2	JN832017
	Tassga	15	30.10; 8.28	*Buthus* spec.	22-II-2009	9-5-1	JN831978
						9-5-2	JN831981
	S Tassoumate	16	30.33; 8.15	*B. elmoutaouakili*	11-III-2009	9-72-1	JN832026
						9-72-3	JN832027
	Tassoumate	17	30.35; 8.15	*B. elmoutaouakili*	11-III-2009	9-71-1	JN832023
						9-71-2	JN832024
	SW Taliouine	18	30.22; 8.09	*B. elmoutaouakili*	11-III-2009	9-70-1	JN831997
						9-70-3	JN831998
	Igherm	19	30.06; 8.70	*Buthus* spec.	22-II-2009	9-4-1	JN832019
**G**	75 km W Tazenakht	20	30.30; 7.52	*B. malhommei*	10-III-2009	9-67-1	JN885945
						9-67-4	JN885946
						9-67-5	JN885947
	W Tazenakht	21	30.27; 7.39	*B. malhommei*	10-III-2009	9-66-2	JN885943
						9-66-3	JN885944
	Tazenakht	22	30.28; 7.22	*B. malhommei*	10-III-2009	9-65-2	JN885941
						9-65-5	JN885942
	Tazenakht	23	30.36; 7.16	*Buthus* spec.	28-II-2009	9-22-1	JN885906
						9-22-2	JN885907
	Agouine	24	31.05; 7.17	*Buthus* spec.	3-III-2009	9-30-1	JN885912
						9-30-2	JN885913
	Iriri	25	30.56; 7.13	*B. albengai*	28-II-2009	9-24-1	JN885908
						9-24-2	JN885909
**H**	Aid Ben Haddou	26	31.05; 7.08	*B. albengai*	1-III-2009	9-25-1	JN885910
						9-25-2	JN885911
	Ouarzazate	27	30.55; 6.51	*B. malhommei*	9-III-2009	9-62-2	JN885939
						9-62-3	JN885940
**I**	Tata	28	29.41; 8.09	*Buthus* spec.	25-II-2009	9-15-1	JN885900
						9-15-2	JN885901
	Ouarzazate	29	30.33; 7.09	*B. draa*	28-II-2009	9-21-1	JN885904
						9-21-2	JN885905
	Ouarzazate	30	30.51; 6.51	*Buthus* spec.	4-III-2009	9-31-4	JN885914
	SE Ouarzazate	31	30.48; 6.44	*Buthus* spec.	4-III-2009	9-32-4	JN885915
	Ait Sarin	32	30.44; 6.38	*Buthus* spec.	4-III-2009	9-33-1	JN885916
						9-33-2	JN885917
	Rebat	33	30.44; 6.26	*B. draa*	5-III-2009	9-34-3	JN885918
						9-34-4	JN885919
	Agdz	34	30.41; 6.26	*B. draa*	5-III-2009	9-Agdz-1	JN885956
						9-Agdz-2	JN885957
**J**	Fumzguid	35	30.04; 6.52	*Buthus* spec.	27-II-2009	9-20-1	JN885902
						9-20-2	JN885903
**K_a**	Sidi-Flah	36	31.00; 6.27	*B. malhommei*	9-III-2009	9-61-1	JN885937
						9-61-2	JN885938
	Skoura	37	31.01; 6.29	*B. malhommei*	9-III-2009	9-60-1	JN885936
	Skoura	38	31.08; 6.21	*B. malhommei*	9-III-2009	9-59-5	JN885935
	M'Gouna	39	31.14; 6.06	*B. malhommei*	9-III-2009	9-58-1	JN885934
	El-Kelaa	40	31.17; 6.08	*B. malhommei*	8-III-2009	9-57-5	JN885933
	Boumalne de Dades	41	31.22; 5.57	*B. malhommei*	8-III-2009	9-53-1	JN885929
						9-53-2	JN885930
	Juniter	42	31.22; 5.47	*Buthus* spec.	7-III-2009	9-52-1	JN885928
	Emsoudar Aitsdrat	43	31.27; 5.58	*B. malhommei*	8-III-2009	9-54-1	JN885931
						9-54-3	JN885932
**K_b**	N'Kob	44	30.51; 5.51	*B. draa*	4-III-2009	9-35-1	JN885920
	Tazzarine	45	30.50; 5.30	*B. draa*	5-III-2009	9-38-2	JN885921
	N Alnif	46	31.10; 5.13	*B. draa*	5-III-2009	9-40-2	JN885922
**K_c**	Tinerhir	47	31.27; 5.36	*B. albengai*	7-III-2009	9-51-2	JN885927
	Asselab	48	31.32; 4.40	*B. draa*	6-III-2009	9-44-2	JN885923
						9-44-3	JN885924
	Ait El Farsi	49	31.22; 5.17	*B. draa*	6-III-2009	9-47-1	JN885925
						9-47-5	JN885926
	Oulad Driss	50	29.49; 5.38	*Buthus* spec.	16-III-2009	9-91-3	JN885952
						9-91-6	JN885953
**L**	Draa Valley	51	30.00; 5.32	*Buthus draa*	16-III-2009	9-A14-1	JN885954
						9-A14-4	JN885955
**Out**	Zagora	52	33.85; 6.29	*Androctonus spec.*	17-III-2009	9-88-1	JN885949
	Mesrate	53	30.03; 5.36	*Androctonus* spec.	16-III-2009	9-89-1	JN885950
						9-89-2	JN885951

**Figure 1:**
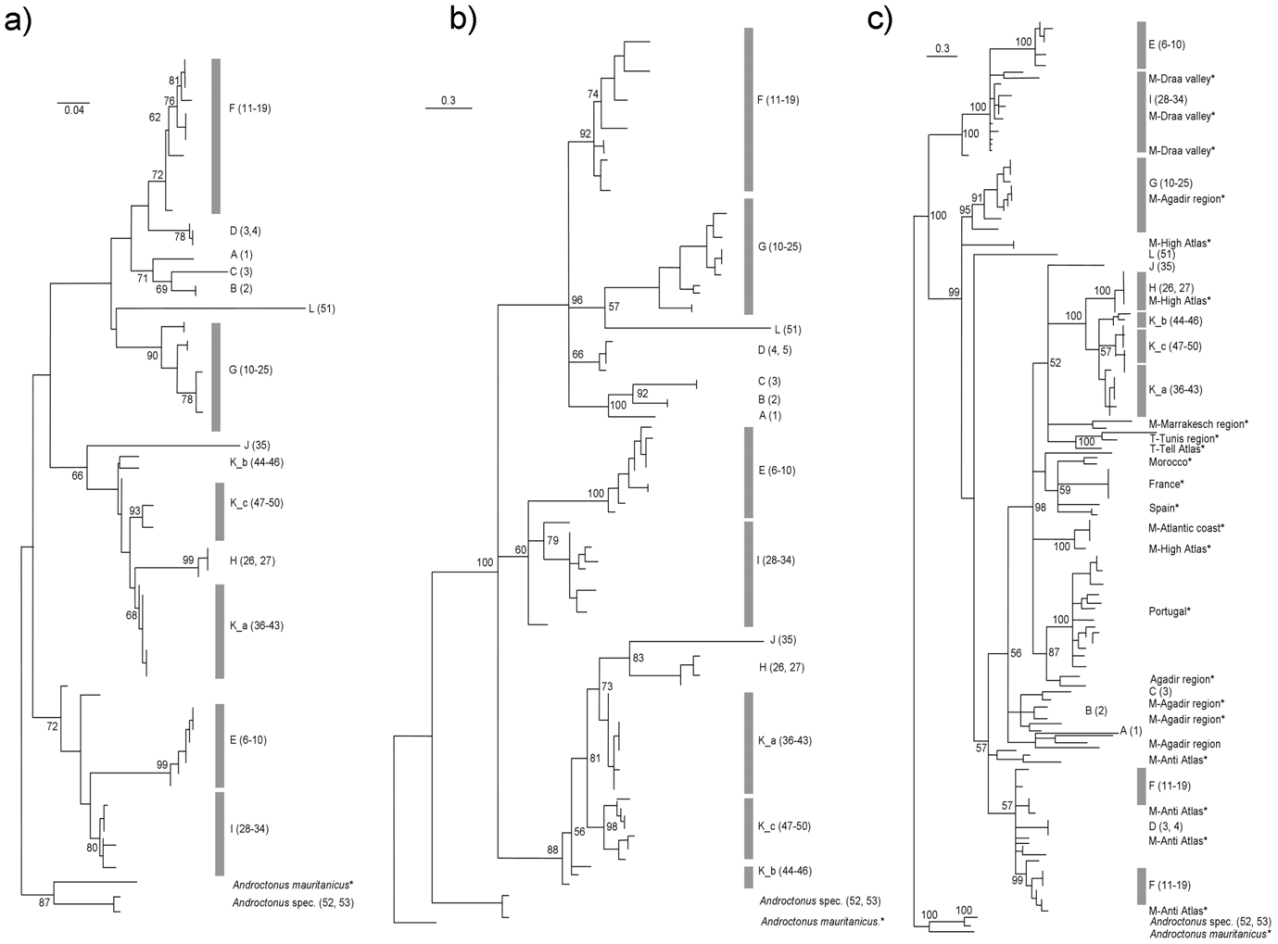
Maximum Likelihood (ML) (a) and Bayesian Inference (BI) tree (b) rooted with *Androctonus mauretanicus* (N = 1) and *Androctonus* spec. (N = 3) as outgroups. Numbers at nodes refer to bootstrap support for ML analyses and posterior probabilities for BI. Figure 1c shows a Bayesian Inference tree including all *Buthus* COI sequences available from genbank (sequences taken from genbank indicated by stars). For detailed information of sequences taken from genbank see appendix SI.

Given are the respective genetic groups, the locality names, a running number for each site (coinciding with figure 1), GPS coordinates of localities, morphological species assignment, date of captures and Genbank accession numbers.

**Table 2:** Fst values between populations estimated under the K2P substitution model; significance values (bold, α = 0.05) were assigned using 1000 permutations.

	A	B	C	D	E	F	G	H	I	J	K	L
A												
B	1.000											
C	0.975	0.984										
D	0.973	0.978	0.976									
E	0.881	**0.903**	**0.901**	**0.884**								
F	0.680	**0.768**	**0.801**	**0.706**	**0.827**							
G	0.754	**0.772**	**0.768**	**0.801**	**0.832**	**0.722**						
H	0.957	0.971	0.962	**0.963**	**0.887**	**0.825**	**0.819**					
I	0.813	**0.842**	**0.832**	**0.821**	**0.750**	**0.789**	**0.801**	**0.841**				
J	1.000	1.000	0.991	0.982	**0.902**	**0.834**	**0.823**	0.962	**0.852**			
K	0.797	**0.832**	**0.815**	**0.821**	**0.809**	**0.809**	**0.804**	**0.712**	**0.768**	**0.797**		
L	1.000	1.000	0.992	0.982	**0.906**	**0.837**	**0.814**	**0.972**	**0.851**	1.000	**0.840**	

The second dataset included 84 additional sequences obtained from genbank (appendix SI). In total this more comprehensive dataset consisted of 179 sequences with 383 bp each. This included 148 variable sites (38.6%), 99 of which were parsimony informative. Many of these sites were uninformative due to a relatively large amount (60 sites) of missing data in genbank sequences.

Reconstructed trees indicate the presence of 12 distinct *Buthus* lineages, which were recovered in all analyses (Fig. 1a–c). However, the relationships among groups differed depending on the tree reconstruction method. This is also reflected in the low statistical support for among group relationships. The support for most groups is high. The genetic patterns strongly coincide with the geographical distribution of the respective sampling locations. Lineages that occur sympatrically (K; most probably H/G/I, Figure 1) are genetically well separated in the trees, while genetically closer groups are all strictly allopatric or parapatric (e.g. I/E; H/K). The distributional patterns of most of the genetic clusters conform to the orographic pattern of the region (e.g. along valleys, southern or northern slopes of mountain systems).

The comprehensive analyses of genbank data together with our own dataset generally yielded geographically meaningful clusters. The European samples represent two monophyletic groups, one in Portugal and one in France also including Spain. These clades likely correspond to the recently described species, *Buthus montanus* Lourenço & Vachon, 2004 and *Buthus ibericus* Lourenço & Vachon, 2004. However, the European *Buthus* are polyphyletic, yet restricted to one branch of the entire tree. They also make the North African *Buthus* paraphyletic (see Figure 1c).

The 12 lineages A through L do not match with current taxonomy (as reflected by morphology based identification keys): (i) *B. elmoutaouakili* Lourenço & Qui, 2006 is found in six lineages, *B. malhommei* Vachon, 1949 in four, *B. albengai* Lourenço, 2003 in two and *B. draa* Lourenço & Slimani, 2004 in four. None of the four species is monophyletic for our marker gene. (ii) Several lineages are composed of different species: four of the five lineages with ten or more individuals analysed contain two to three species; even lineage H (four individuals) includes two different morpho-types.

## Discussion

The two tree hypotheses (ML and BI) constructed from our original dataset distinguish 12 genetic lineages. One of these clusters (lineage K) is further structured into three sub-clades. The genetic clusters exhibit a clear geographic pattern with all the lineages being confined to relatively restricted areas. None of the lineages is widely distributed. Most lineages are separated from each other by geographic barriers such as high mountain ranges or steep valleys. Only some basal lineages show range overlap (e.g. K) while all younger groups are strictly allopatric or parapatric (e.g. the three sub-groups of K). Four groups (A–D) are situated around the western and north-western parts of the High Atlas. One group is located in the westernmost Antiatlas (E), three others at the northern foothills of these mountains (F–H) and one at the southern foothills of the Antiatlas (J). The largest cluster (K) is distributed south of the High Atlas, east of the Draa valley, and splits further into three subclades, which form distinct strictly parapatric groups following the orographic structures of this region (e.g. along the Dades valley or restricted to the Jebel Sahro). Another cluster (I) extends from the Draa valley to the south western edge of the Antiatlas, probably including parts of the distribution ranges of clade G and H.

### Barriers and retreats

The geographic distribution of the obtained genetic clusters strongly supports significant impact of orographic structures as dispersal barriers. The extant distribution of *Buthus* scorpions mostly does not exceed elevations higher than 2500 m asl [23], and individuals show low dispersal ability. Thus, high mountain ranges likely acted (and act) as strong barriers to gene flow resulting in genetically distinct clusters found within the Atlas Mountains. This is reflected in our data by fine structured genetic lineages of the sampled *Buthus* individuals. A similar pattern was observed for the same taxonomic group using various molecular markers such as allozymes, nuclear and mitochondrial DNA all of which showed significant genetic differentiation patterns even among local populations [24], [25]. Similar results were found for other organisms with low to moderate mobility [7]. These extremely sedentary species apparently diverged into several narrowly distributed genetic clusters, restricted to the slopes of mountains and river valleys (e.g. the Ourika and Draa). These lineages are found at different altitudes and might be adapted to various ecological niches. We assume that all actual distribution ranges of *Buthus* lineages also include their last centre of differentiation. Thus, our study area may comprise 12 allopatric refugia during the last ice age revealing a much more fine grained biogeographical sub-structure than previously observed in the Maghreb e.g. [7]–[9]. Consequently, the biogeographical structuring of *Buthus* in Morocco is much more complex than the structures observed in other Mediterranean sub-centres [26], most probably as a consequence of strong gene flow barriers represented by high mountain ranges and a more constant climatic situation in North Africa compared to the northern part of the Mediterranean. The combination of our data with previously analysed *Buthus* individuals (Figure 1c) underlines this superposition of the Maghreb region as a speciation and differentiation centre: The European *Buthus* are polyphyletic, but restricted to one branch of the entire tree and make the North African *Buthus* paraphyletic. This may be indicative of multiple colonisation events from North Africa to Europe.

### The trigger of genetic differentiation

This distribution of genetic lineages supports an allopatric differentiation scenario as opposed to sympatric speciation processes [27]: The lineages originating from the oldest vicariance events are most strongly differentiated from each other. Perhaps this long period of time elapsed since the onset of differentiation allowed for the evolution of characteristics enabling sympatric occurrence of representatives of different genetic groups. Furthermore, the close geographic proximity among the well differentiated lineages G, H and I suggests some areas of range overlap among them. If reproductive barriers are in effect, species might even occur in sympatry without niche differentiation when resources are abundant. Thus, the time elapsed since the onset of the evolution of these lineages must have been sufficient that neither the inter-lineage competition for space nor the strong stenotopy of *Buthus* scorpions has been able to prevent the geographic intermixing of these lineages. However, differentiation among them still might not be sufficient for syntopic occurrences so that these groups exclude each other at each single locality. Such a scenario of competitive exclusion would reinforce a strictly geography-dependent distribution pattern of lineages.

### Discordance between morphospecies and COI phylogeny

In contrast to the stringent geographic pattern, our findings are not congruent with the current taxonomic classification. The different species identified based on morphological characters often cluster within different genetic lineages which, in turn, often comprise more than one species. In addition, many specimens could not be identified due to incomplete or doubtful identification keys, and a lack of diagnostic traits. Hence, the current taxonomy of North African *Buthus* species is in need of revision. Additional diagnostic characters must be identified and new species keys be developed. However, to actually define the status of the detected genetic lineages, further morphological, behavioural and ecological studies are necessary. Besides, it is well known that gene trees and species trees may differ, e. g. due to shared ancestral polymorphisms, incomplete phylogenetic lineage sorting and introgression [28]. Evidence of recombination of mtDNA has been found in *Buthus*, which could also influence our results [29]. Taking our results as an exclusive basis for taxonomical conclusions would therefore doubtlessly be premature. In future studies, the addition of nuclear markers is indispensable to solve the question how prominent hybridization and introgression are among these *Buthus* lineages and species.

## Materials and Methods

### Sampling and identification

**Figure 2:**
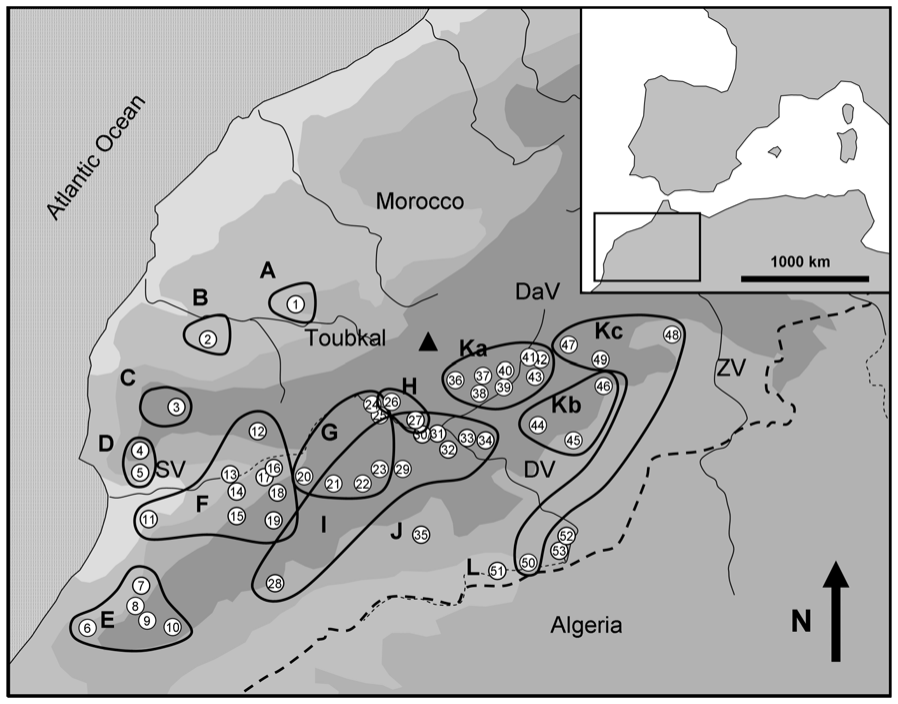
Geographical distribution of all sampling sites and the 12 genetic groups and three subgroups around the Atlas Mountains in Morocco. Numbers of sampling sites coincide with numbers given in Table 1. Letters of the groups correspond with Figure 1. The geographic coordinates of sampling sites are given in Table 1. Abbreviations: SV: Souss valley, DV: Draa valley, DaV: Dades valley, ZV: Ziz valley. Country border between Morocco and Algeria is indicated by a dotted line.

Most specimens were collected at daylight under stones. Overnight collections were facilitated by black light as all scorpions fluoresce in UV light due to a specific protein in their exoskeleton [30]. Collected specimens were stored in absolute ethanol until DNA extraction. All sampling sites are shown in Figure 2 and listed in Table 1 which also gives details on the sampling date and location. Species were identified with keys and additional species descriptions [10], [12], [31]–[33] based on typical morphological characters used for scorpion identification [cf. 32]–[33]. All species identifications were performed by the same author (IS).

### PCR and sequencing

For all individuals, DNA was isolated from leg, caudal segment or telson muscle tissue using the Qiagen DNeasy kit with standard protocols for tissue samples. A 452 bp fragment of the mitochondrial Cytochrome Oxidase I (*COI*) gene was amplified using standard PCR procedures with the following primers: forward 5′ GGT CAA CAA ATC ATA AAG ATA TTG G 3′, reverse 5′ TAA ACT TCA GGG TGA CCA AAA AAT CA 3′ [34]. PCRs were performed in 20 µl volumes: 10 µl Mastermix (Thermozyme), 0.2 µl of each Primer (1 µM), 4.6 µl PCR grade water and 5 µl DNA template. The cycle programme comprised an activation step at 94°C for 4 min, followed by 40 cycles of 30 sec denaturation at 94°C, 30 sec annealing at 45°C and 1 min elongation at 72°C. Cycling was terminated by a final extension step at 72°C for 10 min. Amplicons were subsequently purified and sequenced in both directions on an automated sequencer (3730xl DNA Analyzer; Applied Biosystems, Carlsbad, CA, USA) at the University of Kiel. All sequences are deposited at the NCBI genbank (appendix SI).

### Data analysis

We included 91 sequences of *Buthus*, each 452 bp in length, in our analyses. Four sequences of the Buthid genus *Androctonus* served as outgroup (one sequence from *A. mauretanicus* obtained from genbank: accession JF820097 and three sequences from *Androctonus* spec. (09-88-1; 09-89-1; 09-89-2)). Sequences were inspected and aligned using Geneious 5.0.3 [35].

General alignment statistics (diversity indices, etc.) were calculated using DNAsp v. 5.10 [36]. Estimates of population divergence were calculated as pairwise Fst under a Kimura2P substitution model in Arlequin v.3.5.1.2. [37]. Populations were defined a posteriori according to our phylogenetic results. Significance of Fst values was tested using 1000 permutations and a significance level of 0.05.

The best fitting model of sequence evolution for phylogenetic tree reconstruction was chosen using MrModeltest 2.3 [38] in PAUP 4.0 b10 [39] and was determined to be the GTR+I+G. Maximum Likelihood (ML) trees were constructed using MEGA version 5.01 [40]; 1000 bootstrap replicates were performed to obtain a statistical measure for branch support (Figure 1a). Additionally, we used the Bayesian algorithm implemented in MrBayes 3.1.2 [41] to obtain a second phylogenetic inference and evaluate the stability of our tree reconstruction (Figure 1b). Using the obtained substitution model we ran the Markov chain Monte Carlo algorithm with four chains and two independent runs for 20 million generations. Trees were sampled every 2,000 generations; the first 10% of generated trees were discarded as burn-in as recommended by Tracer v1.5 [42]. The remaining trees were used to generate a consensus tree.

In a second analysis, we included 84 sequences from *Buthus* scorpions obtained from genbank (see appendix SI) resulting in a total of 175 ingroup sequences. However, due to only partial overlap the alignment was reduced to 383 bp in length. Bayesian analyses of this dataset were performed as described above. While these additional analyses provide further insights into geographic structuring of genetic divergence, they do not help in evaluating species identity and monophyly (Figure 1c). When these sequences were submitted to Genbank, the vast majority of Moroccan *Buthus* species known today had not yet been described and most Moroccan *Buthus* were characterized as subspecies of *B. occitanus* (*B. o. occitanus* – Europe, *B. o. mardochei* – Morocco, *B. o. tunetanus* – Tunisia) [31].

## Supporting Information

Appendix SI **DNA sequences obtained from NCBI genbank.**
(DOC)
