# Supplementary material for: Microallopatry Caused Strong Diversification in Buthus scorpions (Scorpiones: Buthidae) in the Atlas Mountains (NW Africa)
Source: PLoS One. 2012 Feb 27;7(2):e29403. doi: 10.1371/journal.pone.0029403 (PMC3287997; doi:10.1371/journal.pone.0029403)
Supplement: Appendix SI — DNA sequences obtained from NCBI genbank. (DOC) [file pone.0029403.s001.doc]

**Appendix SI:** DNA sequences obtained from NCBI genbank. Given are genbank accession number, species, region, locality and reference. Abbreviations: T: Tunisia, M: Morocco, P: Portugal, F: France, S: Spain.

| **Code** | **Species** | **Country-Locality, Region** | **Reference** |
| --- | --- | --- | --- |
| FJ198055 | *B.* spec. | M-n.a. | [16] |
| FJ198056 | *B.* spec. | M-n.a. | [16] |
| AJ506919 | *Androctonus australis* | T-Nefta, Tell Atlas | [17] |
| AJ506869 | *B. atlanti* | M-Essaouira | [17] |
| AJ506870 | *B. atlantis* | M-Pointe Immesouane, Atlantic cosast | [17] |
| AJ506871 | *B. atlantis* | M-Tamri, Atlantic coast | [17] |
| AJ506872 | *B. atlantis* | M-Aourir, Atlantic coast | [17] |
| AJ506873 | *B. occitanus mardochei* | M-Taroudannt, Antiatlas | [17] |
| AJ506874 | *B. occitanus mardochei* | M-Tioute, Antiatlas | [17] |
| AJ506875 | *B. occitanus mardochei* | M-Tioute, Antiatlas | [17] |
| AJ506876 | *B. occitanus mardochei* | M-Tafraoute, Antiatlas | [17] |
| AJ506877 | *B. occitanus mardochei* | M-Adai, Antiatlas | [17] |
| AJ506878 | *B. occitanus mardochei* | M-Irherm, Antiatlas | [17] |
| AJ506879 | *B. occitanus mardochei* | M-Irherm, Antiatlas | [17] |
| AJ506880 | *B. occitanus mardochei* | M-Talmest, Agadir region | [17] |
| AJ506881 | *B. occitanus mardochei* | M-Ounara, Agadir region | [17] |
| AJ506882 | *B. occitanus mardochei* | M-Ounara, Agadir region | [17] |
| AJ506883 | *B. occitanus mardochei* | M-Tamanar, Agadir region | [17] |
| AJ506884 | *B. occitanus mardochei* | M-Immousser valley, Agadir region | [17] |
| AJ506885 | *B. occitanus mardochei* | M-Tazenakht, Agadir region | [17] |
| AJ506886 | *B. occitanus mardochei* | M-Tazenakht, Agadir region | [17] |
| AJ506887 | *B. occitanus mardochei* | M-Bou Azzer, Draa valley | [17] |
| AJ506888 | *B. occitanus mardochei* | M-Ait Saoun, Draa valley | [17] |
| AJ506889 | *B. occitanus mardochei* | M-Ait Saoun, Draa valley | [17] |
| AJ506890 | *B. occitanus mardochei* | M-Zagora, Draa valley | [17] |
| AJ506891 | *B. occitanus mardochei* | M-Tissinnt, Draa valley | [17] |
| AJ506892 | *B. occitanus mardochei* | M-Taddert, High Atlas | [17] |
| AJ506893 | *B. occitanus mardochei* | M-Taddert, High Atlas | [17] |
| AJ506894 | *B. occitanus mardochei* | M-Agouim, High Atlas | [17] |
| AJ506895 | *B. occitanus mardochei* | M-Agouim, High Atlas | [17] |
| AJ506896 | *B. occitanus mardochei* | M-Ouarzazate, High Atlas | [17] |
| AJ506897 | *B. occitanus mardochei* | M-Ouarzazate, High Atlas | [17] |
| AJ506898 | *B. occitanus mardochei* | M-TizinTichka, high Atlas | [17] |
| AJ506899 | *B. occitanus mardochei* | M-TizinTichka, High Atlas | [17] |
| AJ506900 | *B. occitanus mardochei* | M-Igherm'n Ougdal, High Atlas | [17] |
| AJ506901 | *B. occitanus mardochei* | M-Tikirt, High Atlas | [17] |
| AJ506902 | *B. occitanus mardochei* | M-Tikirt, High Atlas | [17] |
| AJ506903 | *B. occitanus mardochei* | M-Cabod de Aguas | [17] |
| AJ506904 | *B. occitanus mardochei* | M-Dar Boazza, Casablanca region | [17] |
| AJ506905 | *B. occitanus occitanus* | F-St. Pons Abbey, Montpellier region | [17] |
| AJ506906 | *B. occitanus occitanus* | F-St. Pons Abbey, Montpellier region | [17] |
| AJ506907 | *B. occitanus occitanus* | F-Narbonne | [17] |
| AJ506908 | *B. occitanus occitanus* | F-Banyuls | [17] |
| AJ506909 | *B. occitanus occitanus* | F-Banyuls | [17] |
| AJ506910 | *B. occitanus occitanus* | S-Escala | [17] |
| AJ506911 | *B. occitanus occitanus* | P-Mertola | [17] |
| AJ506912 | *B. occitanus occitanus* | P-Mertola | [17] |
| AJ506913 | *B. occitanus paris* | M-Ait Ourir, Marrakech region | [17] |
| AJ506914 | *B. occitanus paris* | M-Demnate, Marrakech region | [17] |
| AJ506915 | *B. occitanus tunetanus* | T-Tazoghrane, Tunis region | [17] |
| AJ506916 | *B. occitanus tunetanus* | T-Tozeur, Tell Atlas | [17] |
| AJ506917 | *B. occitanus tunetanus* | T-Moulares, Tell Atlas | [17] |
| AJ506918 | *B. occitanus tunetanus* | T-Tamerza, Tell Atlas | [17] |
| AJ507584 | *B. occitanus mardochei* | M-Igherm'n Ougdal, High Atlas | [17] |
| AJ514323 | *B. atlantis* | M-Pointe Immesouane, Atlantic coast | [17] |
| AJ517182 | *B. occitanus occitanus* | S-Cadiz region, Picacho | [17] |
| AJ517183 | *B. occitanus occitanus* | S-Cadiz region, Picacho | [17] |
| AJ517184 | *B. occitanus occitanus* | S-Malaga, Ubrigue, Benaocaz | [17] |
| AJ517296 | *B. occitanus occitanus* | S-Almeria | [17] |
| GQ168519 | *B. ibericus* | P | [18] |
| GQ168520 | *B. ibericus* | P | [18] |
| GQ168521 | *B.* spec. | P | [18] |
| GQ168522 | *B.* spec. | S | [18] |
| GQ168523 | *B.* spec. | S | [18] |
| GQ168524 | *B. occitanus* | S | [18] |
| GQ168525 | *B. ibericus* | P | [18] |
| GQ168526 | *B.* spec. | P | [18] |
| GQ168527 | *B. ibericus* | P | [18] |
| GQ168528 | *B. ibericus* | P | [18] |
| GQ168529 | *B. ibericus* | P | [18] |
| GQ168530 | *B. ibericus* | P | [18] |
| GQ168531 | *B. ibericus* | P | [18] |
| GQ168532 | *B. ibericus* | P | [18] |
| GQ168533 | *B. ibericus* | P | [18] |
| GQ168534 | *B.* spec. | P | [18] |
| GQ168535 | *B. ibericus* | P | [18] |
| GQ168536 | *B. ibericus* | P | [18] |
| GQ168537 | *B. ibericus* | P | [18] |
| GQ168538 | *B. Ibericus* | P | [18] |
| GQ168539 | *B. Ibericus* | P | [18] |
| GQ168540 | *B. ibericus* | S | [18] |
| GQ168541 | *B.* spec. | S | [18] |
| GQ168542 | *B. ibericus* | S | [18] |
| JF820096 | *B.* spec. | M | [19] |
| JF820097 | *Androctonus mauritanicus* | M | [19] |
